# Supplementary material for: Influence of alcohol-serving venue characteristics on yield of HIV status-neutral screening in rural East Africa
Source: PLOS Glob Public Health. 2026 Jul 27;6(7):e0005053. doi: 10.1371/journal.pgph.0005053 (PMC13405065; doi:10.1371/journal.pgph.0005053)
Supplement: S1 Table — (DOCX) [file pgph.0005053.s001.docx]

**S1 Table.** Venue characteristics associated with likelihood of linking to facility-based screening among participants who received recruitment cards; unadjusted odds ratios.

| **Venue characteristics** | **OR** | **95% CI** | **p-value** |
| --- | --- | --- | --- |
| Formal bar (vs. informal venue) | 0.83 | 0.67 - 1.02 | 0.082 |
| Venue serving commercial brew only | 0.46 | 0.37 - 0.58 | <**0.001** |
| Venue serving both local and commercial brew | 0.50 | 0.42 - 0.60 | <**0.001** |
| Number of rooms (per 1 room increase) | 0.80 | 0.75 - 0.85 | <**0.001** |
| Outdoor venues | 1.79 | 1.38 - 2.33 | <**0.001** |
| Mixed but predominantly indoor | 1.02 | 0.85 - 1.23 | 0.817 |
| Mixed but predominantly outdoor | 1.94 | 1.51 - 2.49 | <**0.001** |
| Evenly mixed indoor and outdoor | 0.82 | 0.62 - 1.11 | 0.197 |
| Patrons per weekday (per 1 patron increase) | 0.99 | 0.98 - 0.99 | <**0.001** |
| Patrons per weekend (per 1 patron increase) | 1.00 | 0.99 - 1.01 | 0.722 |
| Venue workers (per 1 worker increase) | 0.90 | 0.86 - 0.94 | <**0.001** |
| Venue with barmaid(s) | 0.88 | 0.84 - 0.93 | <**0.001** |
| Rooms for sex work | 0.40 | 0.33 - 0.50 | <**0.001** |
| Condoms available on site | 0.55 | 0.46 - 0.65 | <**0.001** |
