# Supplementary material for: Influence of alcohol-serving venue characteristics on yield of HIV status-neutral screening in rural East Africa
Source: PLOS Glob Public Health. 2026 Jul 27;6(7):e0005053. doi: 10.1371/journal.pgph.0005053 (PMC13405065; doi:10.1371/journal.pgph.0005053)
Supplement: S2 Table — (DOCX) [file pgph.0005053.s002.docx]

**S2 Table**. Univariate (unadjusted) associations between alcohol-serving venue characteristics and HIV screening outcomes among adults recruited from alcohol-serving venues, including: a) newly diagnosed HIV, b) known HIV and out-of-care, and c) increased risk of HIV, if HIV uninfected.

| **Venue characteristics** | **a) Newly diagnosed HIV**  OR (95% CI)  p-value | **b) Known HIV+, out-of-care**  OR (95% CI)  p-value | **c) Increased HIV risk, if HIV-**  OR (95% CI)  p-value |
| --- | --- | --- | --- |
| Formal bar (vs. informal venue) | 1.03 (0.60 - 1.75)  p=0.923 | 0.50 (0.22 - 1.15)  p=0.105 | 1.02 (0.79 - 1.33)  p=0.874 |
| Venue serves commercial alcohol only (vs. locally made alcohol only) | 2.37 (1.37- 4.09)  **p=0.002** | 0.90 (0.46 - 1.77)  p=0.770 | 0.99 (0.76 - 1.30)  p=0.962 |
| Number of rooms (aOR per 1 room increase) | 1.21 (1.01 - 1.45)  **p=0.035** | 1.06 (0.89 - 1.26)  p=0.501 | 1.07 (0.98 - 1.17)  p=0.127 |
| Patrons per weekday (aOR per 1 patron increase) | 1.01 (0.98 - 1.03)  p=0.679 | 1.00 (0.97 - 1.02)  p=0.804 | 1.02 (1.01 - 1.03)  **p=0.001** |
| Patrons per weekend (aOR per 1 patron increase) | 0.99 (0.96 - 1.02)  p=0.684 | 1.00 (0.97 - 1.03)  p=0.979 | 1.01 (0.99 - 1.02)  p=0.336 |
| Venue workers (aOR per 1 worker increase) | 1.02 (0.87 - 1.19)  p=0.815 | 0.85 (0.68 - 1.05)  p=0.134 | 1.08 (1.02 - 1.14)  **p=0.013** |
| Venue barmaid (aOR per 1 barmaid increase) | 1.13 (0.97 - 1.32)  p=0.115 | 0.81 (0.65 - 1.01)  p=0.065 | 1.11 (1.04 - 1.18)  **p=0.001** |
| Rooms for sex work | 2.50 (1.46 – 4.28)  **p=0.001** | 1.22 (0.56 – 2.65)  p=0.615 | 1.20 (0.99 – 1.45)  p=0.067 |
| Condoms available on site | 2.75 (1.72 – 4.39)  **p=<0.001** | 0.59 (0.24 – 1.47)  p=0.257 | 1.21 (1.01 – 1.44)  **p=0.043** |
