# Supplementary material for: Influence of alcohol-serving venue characteristics on yield of HIV status-neutral screening in rural East Africa
Source: PLOS Glob Public Health. 2026 Jul 27;6(7):e0005053. doi: 10.1371/journal.pgph.0005053 (PMC13405065; doi:10.1371/journal.pgph.0005053)
Supplement: Attachment — Submitted filename: Comments for Manuscript Number PGPH-D-25-01876.docx [file pgph.0005053.s003.docx]

**Reviewer Recommendation and Comments for Manuscript Number PGPH-D-25-01876**

Many congratulations to the authors of this paper. Without a doubt, a lot of work went into this. I appreciate the details of the report and the use of simple language.

Generally, it is good work and I only have a couple of suggestions.

| s/n | Comments | Recommendations |
| --- | --- | --- |
| 1 | Improve on the methods (procedures) | Consider providing some information about the “Study staff”. Where they were sourced, training, if any. I imagine this also had an effect on results. |
| 2 | Line 136; capturing of biometrics | It might be good to further reiterate the voluntariness of the fingerprint biometric capturing |
|  | Line 113 and 181; Inconsistency in period reported | Align the period for consistency, July 2023 to Dec 2024 or July 2023 to Jan 2025? |
