# Supplementary material for: Influence of alcohol-serving venue characteristics on yield of HIV status-neutral screening in rural East Africa
Source: PLOS Glob Public Health. 2026 Jul 27;6(7):e0005053. doi: 10.1371/journal.pgph.0005053 (PMC13405065; doi:10.1371/journal.pgph.0005053)
Supplement: Attachment — Submitted filename: Response to Reviewers.docx [file pgph.0005053.s004.docx]

**Response to Reviewers**

Manuscript Reference Number: PGPH-D-25-01876

Title: Influence of alcohol-serving venue characteristics on yield of HIV status-neutral screening in rural East Africa

Thank you once again for taking time to review the manuscript and for providing constructive comments and suggestions. We have edited the manuscript in response to these comments.

Below, are each reviewer’s original comments listed in italics followed by our response to each comment.

**Reviewers' comments:**

**Reviewer #1**:

*Many congratulations to the authors of this paper. Without a doubt, a lot of work went into this. I appreciate the details of the report and the use of simple language. Generally, it is good work and I only have a couple of suggestions.*

**Response**: We thank the reviewer for the congratulations and kind comments.

*1. To improve on the method (procedures), consider providing some information about the “Study staff”. Where they were sourced, and training, if any. I imagine this also had an effect on results.*

**Response**: We now include further information about the study staff in the Methods section, including the cadres, where they were sourced and staffing.

Page 7, paragraph 1, line 108: “We hired all study staff, including research assistants, study nurses, and clinicians, locally in Kenya and Uganda and they received a two-day training on the study protocol and procedures prior to study initiation.”

*2. Line 136; capturing of biometrics. It might be good to further reiterate the voluntariness of the fingerprint biometric capturing*

**Response**: Thank you for this suggestion. We now clarify that fingerprint biometrics were captured voluntarily from participants after obtaining informed consent.

Page 8, paragraph 2, line 144: “Staff obtained digital fingerprint biometric measures voluntarily from participants at enrollment after informed consent, and cross-checked fingerprint biometrics over the course of the study to ensure participants were only enrolled once.”

*3. Line 113 and 181; to address the inconsistency in reported period. Align the period for consistency, July 2023 to Dec 2024 or July 2023 to Jan 2025?*

**Response**: We have corrected the inconsistency and the reporting period has been aligned to July 2023 to December 2024.

Page 11, paragraph 1, line 189: “From July 2023 to December 2024, staff visited these venues to distribute recruitment cards”.

**Reviewer #2**:

*Very promising research in terms of aiding HIV prevention and treatment interventions. Couple of comments for the authors:*

*1. It is good practice to include the proportion of females in your tables e.g. Table 2 and 4.*

**Response**: We now include the proportion of females in Tables 2 (see page 13) and 4 (see page 15).

*2. How were the 8 communities in your study selected? Were any efforts made to ensure that these communities were comparable beyond the population count and communities being in catchment area of a government-associated health center providing HIV care?*

**Response**: We are conducting the OPAL-East Africa study within 8 communities taking part in the intervention arm of an on-going community cluster randomized controlled trial, SEARCH SAPPHIRE (NCT05768763). The communities were selected to be of comparable population size (~10,000 persons/community), within the catchment area of a government-associated health center providing HIV prevention and care, and in rural western Kenya and southwestern Uganda: two high HIV prevalence regions within Kenya and Uganda, respectively.

We now include details on how we selected the eight communities in the manuscript, as follows:

Page 6, paragraph 1, line 94: “The communities were also selected based on participation in the intervention arm of an on-going community cluster randomized controlled trial in East Africa (SEARCH SAPPHIRE trial: NCT05768763), which is offering the SEARCH Dynamic Choice HIV Prevention (DCP) model [15] and person-centered multi-disease care in rural settings with high HIV prevalence in Kenya and Uganda.”

See also, References: page 26, line 441: “15. Kabami J, Kakande E, Chamie G, Balzer LB, Petersen ML, Camlin CS, et al. Uptake of a patient-centred dynamic choice model for HIV prevention in rural Kenya and Uganda: SEARCH SAPPHIRE study. J Int AIDS Soc. 2023 Jul;26 Suppl 1(Suppl 1): e26121.”

*3. It is good practice to spell out an acronym such as AUDIT-C in line 240 when it is used for the first time.*

**Response**: Thank you for the suggestion. We have now written AUDIT-C in full as Alcohol Use Disorders Identification Test - Consumption at first mention.

Page 14, paragraph 2, line 248: “The median Alcohol Use Disorders Identification Test - Consumption (AUDIT-C) score, modified for past three-month alcohol use, was…”

*4. Your study identified some interesting results which I think you could set up to inform future studies and interventions. One of such is the finding that venues with condoms available on site were associated with higher odds of identifying persons with undiagnosed HIV. One would expect the opposite given the crucial role condoms play in HIV prevention. You should offer more insights and discussions into results such as this which are somewhat unexpected. Insights need not be definitive since you may not have the data or literature to back up claims but it could offer plausible reasons into what may be responsible for this observation and set up the need for further inquiry. In this case for example, the increased odds of undiagnosed HIV in venues offering condoms may be due to factors such as availability does not necessarily mean people use them, or it may suggest improper usage. Since people however associate condoms to HIV prevention, they may therefore not take the risk of transmission serious, thus contributing to undiagnosed HIV. If any or all of this is the case, a need for more condom availability as suggested in line 324 may not enough if people are not using them when they are available or use them improperly. This could be further explored in future research - looking at what may be responsible for increased odds for undiagnosed HIV despite the availability of condoms at these venues. Or you can simply look for literature that may have explored this and discuss them.*

**Response**: We thank the reviewer for the thoughtful comment and agree that these findings can help inform future studies and interventions. We now offer more insights and discussion of the unexpected finding that venues offering condoms had increased odds of identifying persons with undiagnosed HIV.

Page 20, paragraph 1, line 328: “Increased HIV risk at venues offering condoms on site may also explain our finding of increased odds of undiagnosed HIV at these venues. Factors such as incorrect or non-usage of condoms, partner resistance, stock-outs and condoms being sold, rather than freely provided, may allow for ongoing HIV transmission despite condom availability.”

Page 20, paragraph 1, line 339: “Further research is needed to better understand the contextual barriers to condom usage in alcohol-serving venues.”
