# Supplementary material for: Influence of alcohol-serving venue characteristics on yield of HIV status-neutral screening in rural East Africa
Source: PLOS Glob Public Health. 2026 Jul 27;6(7):e0005053. doi: 10.1371/journal.pgph.0005053 (PMC13405065; doi:10.1371/journal.pgph.0005053)
Supplement: Attachment — Submitted filename: Response_to_reviewers_auresp_2.docx [file pgph.0005053.s005.docx]

**Response to Reviewers**

Manuscript Reference Number: PGPH-D-25-01876

Title: Influence of alcohol-serving venue characteristics on yield of HIV status-neutral screening in rural East Africa

Thank you once again for taking time to review the manuscript and providing constructive comments, suggestions and the opportunity to address reviewers’ comments. We have edited the manuscript in response to these comments.

Below are each reviewer’s original comments listed in italics followed by our response to each comment.

**Reviewers' comments:**

**Reviewer #3:**

*This is an interesting and well-written report. The authors assess the characteristics of alcohol-serving sites and how they affect the linkage to health care and screening for HIV.*

**Response:** We thank the reviewer for the kind comments and thoughts.

*1. The yield of screening, linkage, newly diagnosed etc... per venue are among the main results of this manuscript. It is so that the tile has the word yield. So, I would suggest to have a figure to show at least overall yield (a bar plot with confidence intervals).*

**Response:** At the reviewer’s suggestion, we have added a bar graph to show overall yield of screening and the results of screening.

Page 15, paragraph 1, line 261: “The overall yield of HIV screening and the results of screening in identifying persons with newly diagnosed HIV, known HIV and out of care and increased HIV risk, are shown in **Fig 2**”.

Page 15, line 267: “**Fig 2. The overall yield of HIV screening and the results of screening in Kenya and Uganda”.**

Percentages represent proportions within each screened group. The numbers in parenthesis (n/N) represent specific screening outcomes (n), including persons with HIV (PWH), new HIV diagnosis and known HIV and out of care among PWH and increased HIV risk among persons without HIV, among the total population evaluated within each category (N).

*2. About the GEE description in the statistical section:
- Make it clear what family was used (binomial?) and what link (logit?) was used
- What working correlation was used here? State this.
- Whether you used naive or robust standard errors.*

**Response:** We thank the reviewer for the opportunity to clarify. We now include a description of the GEE specifications we used for the analysis as follows:

Page 10, paragraph 1, line 174: “In the predictor analyses, we used the binomial family with the logit-link, an independent working correlation matrix to account for clustering by alcohol-serving venue, and obtained robust (sandwich) standard errors.”

*3. Tables 3 and 5: please state somewhere in the caption number of participants*

**Response:** Thank you for the feedback. We now include in the caption number of participants that received recruitment cards in Table 3 and 5 as follows:

Page 14, line 246: “Table 3: Venue characteristics associated with likelihood of linking to facility-based screening among participants (N=9,375) who received recruitment cards, with odds ratios adjusted for age, sex and country”.

Page 16 line 284: “Table 5: Associations between alcohol-serving venue characteristics and HIV screening outcomes among adults recruited from alcohol-serving venues, including: a) newly diagnosed HIV (N=141), b) known HIV and out-of-care (N=78), and c) increased risk of HIV, if HIV uninfected (N= 2,285); with adjusted odds ratios (aOR) controlling for age, sex and country”.

**Reviewer #4:**

*This is a very interesting, relevant, and well-executed manuscript addressing an important public health opportunity for HIV prevention and case-finding in rural East Africa. The study is original, programmatically meaningful, and highly valuable from a public health perspective because it explores how venue-level characteristics can help identify settings where HIV screening, linkage, and prevention interventions may be most impactful.

The revised version has improved substantially. The authors have addressed the main concerns raised in the previous round, particularly by clarifying the role and training of study staff, the voluntary nature of biometric collection, the selection of study communities, the expansion of the acronym AUDIT-C, and the interpretation of the unexpected findings related to condom availability at venues. These revisions strengthen the manuscript.*

*Overall, I believe this paper is suitable for publication after minor revision, as only a few residual issues remain.*

**Response:** We thank the reviewer for the positive feedback, and for recognizing the relevance and contribution of our work. We appreciate the positive comments on the previous revisions made on the manuscript.

*1. Please check internal numerical consistency in the Results section.
In the Results text, the manuscript states: “Of 7,774 participants…” whereas the total number reported throughout the manuscript, including Table 4, is 7,744. This should be corrected for consistency.*

**Response:** The discrepancy in the reported number of participants was due to a typographical error in the Results text. This has now been corrected to 7,744 participants. We have also reviewed all results to ensure consistency throughout the manuscript.

Page 14, paragraph 2, line 250: “Of 7,744 participants, their overall median age was 34 years (interquartile range [IQR]: 26-43 years), 62% were men, median daily was US$2 (IQR: $1-3), 54% were married and living with their spouse, and 91% were venue patrons”.

*2. Please carefully review citation numbering throughout the revised manuscript.
After the addition of the SEARCH SAPPHIRE reference, some in-text citations appear to have shifted incorrectly. For example:
the citation used for the national HIV testing/treatment guidelines appears inconsistent with the revised reference order; the citation attached to the GEE methodology may also be mis numbered; some discussion citations may no longer correspond to the intended references. A full check of in-text citations against the revised reference list is recommended before acceptance.*

**Response:** Thank you for highlighting this. We have now reviewed all in-text citations and cross-checked them against the revised reference list following the addition of the SEARCH SAPPHIRE reference. The citation numbering has been corrected to ensure accuracy and consistency.

*3. Please review the reference list for duplication and ordering errors.
There appears to be duplication of at least one citation related to HIV prevention in alcohol venues. The reference list should be checked carefully to ensure that: duplicate references are removed, all citations are uniquely numbered, and numbering matches the in-text citations throughout the manuscript.*

**Response:** We have reviewed the reference list and removed the duplicate citation, ensured that all references are uniquely numbered, and that the numbering matches the in-text citations throughout the manuscript.

*4. Clarify the handling of missing data in the regression models.
The manuscript notes missingness for some variables, including patron/worker status, marital status, bar area, and condom availability. It would strengthen the Methods if the authors briefly clarified whether the regression models were based on complete-case analysis and, if so, whether the analytic sample varied across models.*

**Response:** We thank the reviewer for the opportunity to clarify. We now clarify how we handled missing data as follows:

Page 10, paragraph 1, line 178; “All analyses were pooled over trial arm and excluded observations with missing data on the relevant predictor; therefore, the number included in the analytic sample varied across regression models. All analyses were conducted using R version 4.4.2.”

*5. Consider slightly tempering the wording of the conclusion.
The conclusion states that certain venue types “should be prioritized” for interventions. Since the analysis is observational, a slightly more cautious phrasing such as “may be prioritized” or “may represent high-yield settings for targeted outreach” would better align with the design and findings.*

**Response:** Thank you for the suggestion. We have revised the conclusion to a more cautious phrasing as the analysis was observational.

Page 22, paragraph 2, line 393: “Overall, our findings suggest that larger venues, and those with rooms for sex work and with condoms available on site, may represent priority settings for enhanced, community-based interventions, both to increase HIV screening engagement and identify patrons/workers in need of HIV prevention and treatment in East Africa.”
