# Supplementary material for: Influence of alcohol-serving venue characteristics on yield of HIV status-neutral screening in rural East Africa
Source: PLOS Glob Public Health. 2026 Jul 27;6(7):e0005053. doi: 10.1371/journal.pgph.0005053 (PMC13405065; doi:10.1371/journal.pgph.0005053)
Supplement: Attachment — Submitted filename: Response_to_reviewers_auresp_3.docx [file pgph.0005053.s006.docx]

**Response to Reviewers**

Manuscript Reference Number: PGPH-D-25-01876

Title: Influence of alcohol-serving venue characteristics on yield of HIV status-neutral screening in rural East Africa

Thank you once again for taking time to review the manuscript and providing constructive comments, suggestions and the opportunity to address reviewers’ comments.

Below are each reviewer’s original comments listed in italics followed by our response to each comment.

**Reviewers' comments:**

***Reviewer #1:***

*Great work. I appreciate the attention to detail on the issues raised by the reviewers.*

**Response:** We thank the reviewer for the kind comments and thoughts.

***Reviewer #3:*** *All comments have been addressed satisfactorily.*

**Response:** We thank the reviewer for the positive feedback.

***Reviewer #4:***

*Dear Authors,
Thank you for submitting the revised manuscript (PGPH-D-25-01876R2) titled "Influence of alcohol-serving venue characteristics on yield of HIV status-neutral screening in rural East Africa" to PLOS Global Public Health.
I have reviewed the revised manuscript and the responses to reviewers. The authors have adequately addressed the majority of the comments raised in the previous round. The manuscript is well-written and the revisions substantially strengthen the work.
Before final acceptance, I recommend the authors verify the following minor outstanding points:*

**Response:** We appreciate the positive comments on the previous revisions made on the manuscript.

1. *Confirm that confidence intervals are displayed in Figure 2, as specifically requested by Reviewer #3.*

**Response:** We thank the reviewer for this important highlight. We have now displayed the confidence intervals in Figure 2.

2. *Provide confirmation that the full citation list has been audited against the in-text references throughout the entire manuscript, including the Discussion and Conclusions sections.*

**Response:** We confirm that the full citation list has been audited against the in-text references throughout the entire manuscript, including the discussion and conclusion sections.
